# Telomere Length in Norway Spruce during Somatic Embryogenesis and Cryopreservation

**DOI:** 10.3390/plants10020416

**Published:** 2021-02-23

**Authors:** Tuija Aronen, Susanna Virta, Saila Varis

**Affiliations:** Natural Resources Institute Finland (Luke), FI-57200 Savonlinna, Finland; sbevirta@gmail.com (S.V.); Saila.Varis@luke.fi (S.V.)

**Keywords:** cryostorage, embryo production capacity, genotypic variation, *Picea abies*, somatic embryogenesis (SE), telomere fragment length

## Abstract

Telomeres i.e., termini of the eukaryotic chromosomes protect chromosomes during DNA replication. Shortening of telomeres, either due to stress or ageing is related to replicative cellular senescence. There is little information on the effect of biotechnological methods, such as tissue culture via somatic embryogenesis (SE) or cryopreservation on plant telomeres, even if these techniques are widely applied. The aim of the present study was to examine telomeres of Norway spruce (*Picea abies* (L.) Karst.) during SE initiation, proliferation, embryo maturation, and cryopreservation to reveal potential ageing or stress-related effects that could explain variation observed at SE process. Altogether, 33 genotypes from 25 families were studied. SE initiation containing several stress factors cause telomere shortening in Norway spruce. Following initiation, the telomere length of the embryogenic tissues (ETs) and embryos produced remains unchanged up to one year of culture, with remarkable genotypic variation. Being prolonged in vitro culture can, however, shorten the telomeres and should be avoided. This is achieved by successful cryopreservation treatment preserving telomere length. Somatic embryo production capacity of the ETs was observed to vary a lot not only among the genotypes, but also from one timepoint to another. No connection between embryo production and telomere length was found, so this variation remains unexplained.

## 1. Introduction

Eukaryotic chromosomes are formed of a single DNA molecule, which terminates in specialized heterochromatin called telomeres [1]. Telomeres consist of repeated DNA sequence that in most of the plant species is a heptanucleotide (TTTAGGG)n. The function of telomeres is to protect chromosomes from degradation and fusion during DNA replication, and therefore in cell divisions, they are especially important for chromosome organization [1]. Cells’ conventional DNA polymerase is, however, not able to fully replicate the linear termini of the chromosomes i.e., telomeres, but they are maintained by a specific enzyme, telomerase. Without telomerase activity, telomeres shorten in each cell division. If only one or a subset of telomeres is shortened below a critical length, replicative cellular senescence may be triggered by DNA damage response (DDR) [2]. In DDR, cell’s own DNA repair machinery misidentifies the natural ends of the chromosomes as damaged DNA and this can further lead to either programmed cell death or in irreversibly arrested proliferation even if the cells stay alive [2].

The progressive telomere shortening takes place in dividing cells not only due to incomplete end-replication problems, but also as consequence of stress caused by various factors e.g., pathogen attack, poor diet, harsh conditions, competition, reproductive effort etc. [3]. Meta-analyses of data from 109 studies show that exposure to stressors was associated with shorter telomeres or higher telomere shortening rate [3]. The underlying mechanism suggested for stress-induced telomere shortening and replicative cell senescence is oxidative damage caused by reactive oxygen species (ROS). The ROS can be produced during increased mitochondrial activity to generate energy to mitigate stress factor [3] and by mitochondrial dysfunction [4].

Telomeres and their connection to cellular senescence and ageing of various organisms have been extensively studied, and there are also some reports on long-living tree species. Originally, Flanary and Kletetschka [5] suggested increased telomere length and telomerase activity contributing to an extended life span in long-living pines. Later studies have given partly contradictory results: Examination of ginkgo trees (*Ginkgo biloba* L.) of up to 1400 years of age [6,7] showed telomere length increasing with age, while in one- to seven-year-old apple trees (*Malus* × *domestica* Borkh., *Malus* × *prunifolia*) and in up to 20-year-old cherry trees (*Prunus* × *yedoensis* Malsum.), no difference in telomere length due to ageing, or between juvenile, non-flowering, and mature parts of a tree were found [8]. In Scots pine (*Pinus sylvestris* L.), Aronen and Ryynänen [9] found no ageing-related change in the telomeres of the 1–200-year-old trees but observed the telomeres of the cambium shorten towards the top of the older trees. The same positional phenomenon was discovered in 80-year-old silver birch trees (*Betula pendula* Roth) [10]. In Scots pine, telomeres were also shown to shorten with increasing level of tissue differentiation, with embryo samples having the longest repeats [9]. The length of telomeric repeats has also been observed to vary among tissue types in ginkgo trees [6] and according to season in ginkgo [7], ash (*Fraxinus pennsylvanica* Mars. var. subintegerrima [Vahl.] Fern), and willow (*Salix matsudana* Koidz.) [11].

There is, however, only limited information on the effect biotechnological tools, such as tissue culture or cryopreservation on plant telomeres despite the fact that conditions within these techniques per se can be argued to act as stress [12], followed by a transition to ex vitro conditions known to be stressful to plants too [13]. Some studies have shown shortening of telomeres in perennial plants, as found in in vitro shoot and callus cultures of deciduous tree silver birch [10], as well as following ex vitro acclimatization of agave (*Agave tequilana* Weber) [13]. In agave, however, temporary shortening of telomeres was found to be resumed and telomere length maintained thereafter, either based on telomerase activity or alternative mechanisms. In the annual plants, barley (*Hordeum vulgare* L.) [14], white cambion (*Silene latifolia* Poir.) [15], and tobacco (N*icotiana tabacum* L.) [16], either lengthening or no change of telomeres during in vitro callus culture was observed.

Tissue culture based on somatic embryogenesis (SE) has become the method of choice for vegetative propagation of conifers, enabling fast and efficient multiplication of specific genotypes with desired characteristics e.g., fast growth, wood quality, or disease resistance traits [17] or ornamental value [18]. The valuable genotypes can be cryopreserved to avoid ageing and are ready for re-multiplication when needed [18,19]. SE has been adopted for production of forest tree plants e.g., in several coniferous species [20,21,22]. In Norway spruce (*Picea abies* (L.) Karst.), long-term research has resulted in several functional protocols published for both SE [23,24,25,26] and cryostorage of embryogenic cultures [27,28], with efforts for automation of propagation process currently going on [29,30]. In Finland, SE of Norway, spruce is being piloted for commercial mass-propagation of forest regeneration material [31]. Despite all this experience gained and broad materials studied, some unexplained variation at the SE propagation exists. This may be due to many genetic and physiological factors affecting SE through complex regulatory networks that still remain partly unknown [32].

The aim of the present study was to examine telomeres of Norway spruce during SE propagation and cryopreservation. Connections between telomere length and SE initiation, somatic embryo production capacity, and recovery from cryostorage were studied to reveal potential ageing or stress-related effects that could help to explain variation observed at SE process.

## 2. Results

### 2.1. SE Initiation

Success of SE initiation varied among the controlled crosses. In 2012, the initiation frequencies were 61.4–100% depending on the cross (Table A1). In 2014, the SE initiation success varied in all the crosses from 30.0 to 93.5%, and in the crosses from which immature zygotic embryos were sampled for telomere length measurements 36.5–77.0%, correspondingly (Table A1).

### 2.2. Recovery from Cryopreservation

When the established embryogenic lines were cryopreserved using preculture on semisolid media with increasing sucrose concentration, PGD mixture as cryoprotectant, and freezing in programmable freezer, 100% of the samples were recovered. The samples taken for telomere studies, representing non-regenerating cryostored ETs, were either cryopreserved using otherwise the same method but freezing in Mr. Frosty containers instead of a programmable freezer (lines 4934, 6375, 4611, 3128), or pretreated in liquid medium and cryoprotected with Me2SO, followed by freezing in a programmable freezer (line 5852).

### 2.3. Embryo Production Capacity

**Figure 1:**
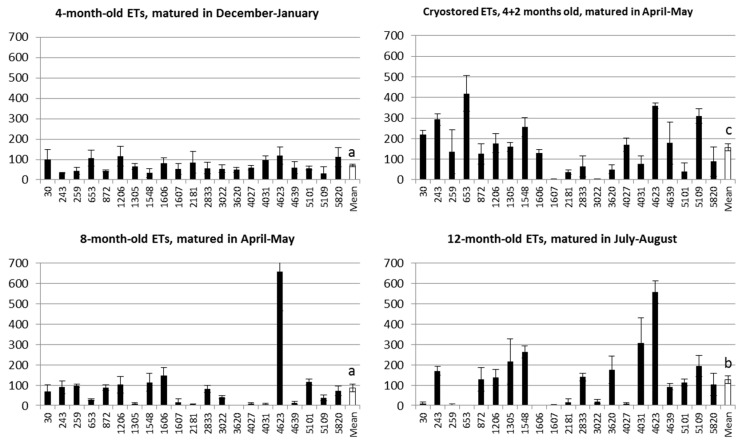
Somatic embryo production of 21 embryogenic lines of Norway spruce following 4, 8, or 12 months in continuous proliferation, or following cryopreservation at age of 4 months, thawing, and further proliferation for 2 months, *n* = 252. Number of embryos produced per gram of fresh weight (gFW) of embryogenic tissue with standard error are shown. The group means that differ significantly from each other (Student–Newman–Keuls multiple range test, *p* < 0.05) are marked by different letters.

Somatic embryo production capacity of the studied materials varied significantly (F = 3.92, *p* = 0.013) among the tested timepoints (Figure 1). On average, the best embryo production was observed in the cryopreserved material, 4 + 2 months in age, matured in the spring. The cryopreserved material was better than the material kept at continuous proliferation, of which the oldest cultures (12 months in age, matured at the summer) yielded more embryos than the younger ones (4 months in age, matured in the winter or 8 months in age, matured in the spring). There was also significant variation among the genotypes (F = 3.17, *p* = 0.000), and a significant interaction between the genotype and the timepoint (F = 4.27, *p* = 0.000) (Figure 1).

### 2.4. Telomere Length in Embryogenic Materials

**Figure 2:**
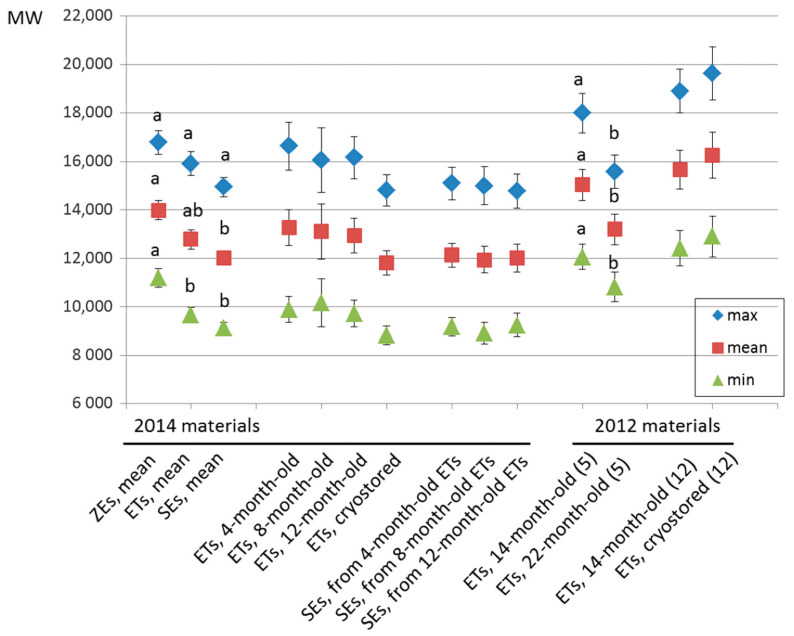
The length of telomeric repeats in the studied Norway spruce materials including immature zygotic embryos (ZEs), proliferating embryogenic tissues (ETs) and mature somatic embryos (SEs): The 2012 (*n* = 33) and 2014 materials (*n* = 85) consisting of different sets of genotypes were studied separately. In the 2012 material, significant differences between 14- and 22-month-old ETs studied with five genotypes are marked by different letters. When examining 12 genotypes of the 2012 material representing ETs continuously proliferated for 14 months or cryostored and proliferated for 14 + 1 months, no significant differences were found. In the 2014 material, no significant differences were found among ETs proliferated for 4, 8, or 12 months or cryostored when compared with each other, nor among SEs derived from different-aged ETs. The 2014 means for sample types (ZEs, ETs, and SEs) that differ significantly (Student–Newman–Keuls multiple range test, *p* < 0.05) from each other are marked by different letters.

**Figure 3:**
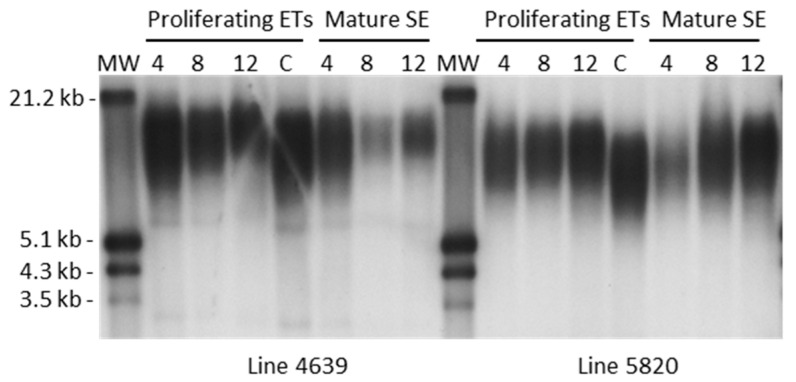
Southern hybridization showing telomere length in two genotypes of Norway spruce: Samples taken from proliferating embryogenic tissues (ETs) after 4, 8, or 12 months of continuous proliferation, or following cryopreservation (C) at age of 4 months, thawing, and further proliferation for 2 months prior to maturation, as well as mature somatic embryos (SE) originating in the ETs of either 4, 8, or 12 months of age are shown.

Telomere length during somatic embryogenesis was studied in 2014 materials consisting of immature zygotic embryos used as explants for SE initiation, proliferating embryogenic cultures with and without cryopreservation, and mature somatic embryos derived from them. When examining the different sample types, some significant differences are seen in the average (F = 3.04, *p* = 0.053) and minimum (F = 4.70, *p* = 0.012) lengths of telomeric repeats, immature zygotic embryos having longer telomeres than somatic embryos (Figure 2). It should be note, however, that zygotic embryo samples consist of different genotypes than embryogenic cultures and somatic embryos derived from them, although all the sample types have the same genetic background i.e., parent trees. When studying variation within embryogenic cultures and mature somatic embryos in the 2014 material, no significant effect of either culture duration (from 4 to 12 months) or cryopreservation on the telomere length is found (Figure 2 and Figure 3).

**Figure 4:**
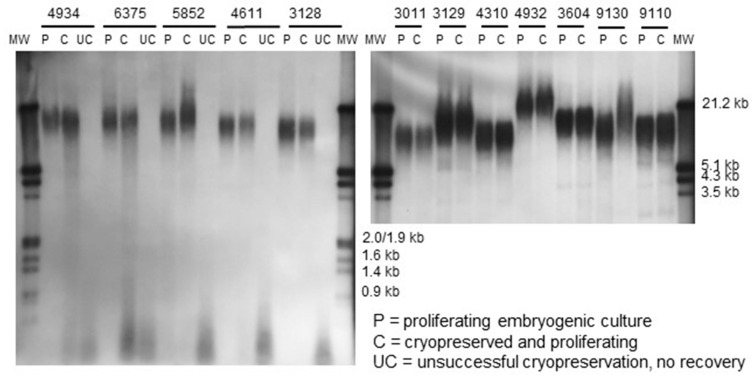
Southern hybridization showing telomere length in proliferating embryogenic tissue of Norway spruce prior to and following cryopreservation: The five genotypes shown on the left panel were cryopreserved both using a method leading to successful regeneration (pretreatment on semisolid media with increasing sucrose concentration, PGD cryoprotectant mixture, and slow cooling in programmable freezer) and by methods resulting in no regeneration (pretreatment in liquid medium and Me2SO as cryoprotectant, or freezing in Mr. Frosty containers at −80 °C), while the seven genotypes shown on the right panel were cryopreserved only using successful method.

In the 2012 material kept in the continuous proliferation for a longer time, from 14 to 22 months, significant difference in telomere length is seen: The minimum (F = 23.43, *p* = 0.004), average (F = 18.30, *p* = 0.009), and maximum length (F = 10.29, *p* = 0.031) of telomeric repeats is shorter in the material cultured for a longer time. No effect of cryopreservation is found in the cases when it was successful i.e., the cultures were proliferating following cryostorage (Figure 2 and Figure 4). If the cryopreservation, however, was not successful resulting in non-regenerating embryogenic cultures, a complete breakdown of the telomeric repeats was observed (Figure 4).

In all the experiments, genotypic differences in telomere length were significant among the ETs and mature embryos derived from them (2014: Maximum length F = 9.21, *p* = 0.000; average length F = 8.76, *p* = 0.000; minimum length F = 6.49, *p* = 0.001. 2012: maximum length F = 13.87, *p* = 0.013; average length F = 13.92, *p* = 0.013; minimum length F = 13.36, *p* = 0.014). The variation was the remarkable, e.g., the average length of telomeric repeats varying in the 2014 material from 8.9 (±SE 0.27) kb to 14.4 (±SE 0.25) kb, and in the 2012 material from 11.5 (± SE 0.68) kb to 23.0 (± SE 0.57) kb, as also easily seen in the Figure 4. In addition, the genotypes originating in the same controlled cross, i.e., full-sibs, were observed to have notable differences in the telomere length: For example the genotype 3128 compared with the 3129, or the 3932 compared with 3934 (Figure 4).

The potential association between telomere length and other studied parameters, such as SE initiation frequency on family level, and somatic embryo production capacity of the genotypes was also analyzed. There was a positive correlation (Pearson r = 0.720, *p* = 0.044) between the maximum length of telomeric repeats in the immature zygotic embryos of the controlled cross (varying from 15.2 to 19.0 kb) and SE initiation frequency (varying from 36.5 to 77.0%), but the corresponding correlations with the average and minimum length of the telomeric repeats (Pearson r = 0.662 and 0.427, respectively) were not significant (*p* = 0.074 and 0.292), respectively. For the embryo production capacity varying a lot among the genotypes and time points, no association with telomere length was found (Pearson r for the maximum, average, and minimum length of the telomeric repeats −0.053, −0.104, and −0.173 with *p* = 0.731, 0.502, and 0.262, respectively).

## 3. Discussion

This study is the first report on telomeres in Norway spruce (*Picea abies* (L.) Karst). In the present spruce material, the average length of telomeric repeats varied from 9 to 23 kb among the genotypes. This is well within the range reported previously for the other coniferous species: In Scots pine (*Pinus sylvestris* L.), true telomeres had an overall mean length of 19.3 kb, also with remarkable genotypic variation, from 15.6 to 23 kb [9]. In other pine species (*P. aristata, P. monticola, P. resinosa, P taeda, P. palustris*, and *P. longaeva*), the shortest telomeric repeats were reported to be 0.5–2.7 kb and the longest ones, 21–57 kb [5]. Genotypic differences in telomere length have been reported also in a deciduous tree, silver birch, in which they were also shown to be consistent over the outdoor and tissue-cultured samples [10].

In the present material, significant differences in telomere length were found—in addition to genotypic differences—in three cases: 1. When comparing different sample types, i.e., immature zygotic embryos, proliferating ETs and mature somatic embryos; 2. when comparing ETs after 14 or 22 months of continuous proliferation; and 3. when comparing the samples of ETs recovered successfully from cryopreservation with the samples showing no recovery. At the same time, there were significant differences in the SE initiation rate among the studied families, and in the somatic embryo production capacity of the studied genotypes and timepoints, and the connections to these observed differences to variation in telomeric repeat length are discussed.

There was a significant correlation between the maximum length of telomeres in the pooled explant material i.e., immature zygotic embryos and the SE initiation rate of the controlled cross, with the ones having the longest telomeres showing highest SE initiation. The correlation between the minimum length of telomeres considered critical for cells’ replication [2] was, however, not significant. It should be remembered that the explant samples consist of numerous zygotic embryos, each representing their own genotype, and that remarkable genotypic differences in telomere length were observed also within the family. The present material is small, eight full-sib families, but the result may indicate that in the crosses with higher SE initiation rate there are more zygotic embryos i.e., genotypes with longer telomeric repeats providing better buffer against stress factors to which they are subjected during SE initiation treatment. Previously, the telomere length of the explants and SE initiation rate has been examined in a small open-pollinated Scots pine material, but no connection was observed [9]. Compared with Norway spruce, however, the SE induction in Scots pine is very difficult, with the mean initiation rates of 0–17.5% achieved in the explants from the studied donor trees [33].

In the SE initiation process used in the present study, the immature cones were collected from trees, seeds extracted from them, surface-sterilized, and finally isolated zygotic embryo was excised from the seed and placed on tissue culture medium [28]. This procedure includes several stress factors, e.g., wounding, harsh conditions, and chemical treatments that are known to cause telomere shortening via reactive oxygen species (ROS) [3]. This stress hypothesis is supported by the fact that a significant difference was found in the minimum length of the telomeres when comparing the explant material i.e., immature zygotic embryos with resulting ETs, the proliferating ETs having shorter telomeres. It should be noted, however, that the explant samples consist of numerous pooled genotypes, and thus genotypic differences when compared with ETs of known genotypes might also have a role. Previously, the telomeres have shown to shorten with increasing level of tissue differentiation, with the embryonal samples having the longest repeats e.g., in Scots pine [9] and barley [14]. In the present material, the observed telomere shortening cannot be related to tissue differentiation, since proliferating ETs, also called proembryogenic masses (PEMs) contain two major cell types: Meristematic cells of the embryonal mass and the embryonal tube cells [34], so the shortening is most probably related to initiation stress. Initiation of other types of tissue culture than SE has resulted on contradictory observations on telomere length: Induction of organogenesis in agave species resulted in telomere lengthening [13], while no change in telomere length was found in tobacco plants produced by organogenesis via callus phase [16].

Following initiation, during proliferation of established ETs and somatic embryo maturation, no changes in telomeres were observed when 2014 materials were studied at several timepoints up to 12 months of in vitro culture. At all the timepoints, however, the mature somatic embryos had shorter telomeres than immature zygotic embryos or ETs containing proembryos. Although this difference with ETs was non-significant, it is consistent with the previous observations of telomeres shortening with differentiation or degree of tissue maturity [9,13,14].

While no differences in telomeres were seen in 2014 materials studied for up to 12 months of culture, significant shortening of telomeres was found in 2012 materials with extended in vitro culture: ETs proliferated continuously for 22 months had shorter telomeres than the ones cultured for 14 months. This is in line with results from another tree species, i.e., tissue-cultured silver birch materials showing prolonged (over four years) in vitro culture causing telomere shortening [10]. On the contrary, in the long-term (one year) cultures of annual barley, telomere lengthening was observed [14].

It is known that under continuous in vitro culture, ETs of conifers may decline or even lose their somatic embryo differentiation ability [35,36]. This phenomenon could be connected to cells’ replicative senescence related to shortening of telomeres below the critical point, and therefore both somatic embryo production capacity and telomere length was studied in 4-, 8-, and 12-month-old ETs of Norway spruce. Within this time series, no change in telomeres was observed in the studied 11 genotypes, while their embryo production varied a lot. Unfortunately, embryo production was not studied in the ETs with proliferation extended to 22 months and showing shortening of the telomeres, but within duration of in vitro culture normally applied in Norway spruce, i.e., up to one year, there was no connection between telomere length and embryo differentiation ability.

In the present material kept at continuous subculture, the somatic embryo production was better in the older, 12-month-old ETs than in the younger ones, of four to eight months in age—opposite to expectations. The best results were, however, received by using ETs recovered from cryopreservation. The cryostored ETs were younger than the ETs from continuous matured at the same time in the spring. If examining the embryo production based on the time point, it is seen that the maturations made in the middle of the winter were less productive than the ones from the spring or summer. In the Norway spruce trees used as donors for ETs, the development of zygotic embryos takes place from the beginning of June to end of August [37], i.e., at the same time than the more productive maturations of the present study. The in vitro grown ETs are not subjected to the environmental factors such as changing photoperiod and temperature naturally controlling the annual growth and development of trees [38]. Molecular mechanisms controlling trees’ annual cycle are still largely unknown in conifers [39] so it cannot be completely excluded that the ETs have an inner clock contributing their embryo production capacity.

In the cryopreservation experiments, a clear connection between telomeric repeats and success of cryopreservation was observed: In the non-recovering ETs, all telomeric signals were less than 1 kb in size, showing dramatic reduction from prior to cryopreservation size varying from 9.7 to 23.5 kb. The cryopreservation method, from which most of the non-recovering samples for the present telomere study were taken, i.e., 2-h freezing in the Mr. Frosty container, has previously been described functional for ETs of Norway spruce, but resulting more often in loss of the ETs than slower freezing in a programmable freezer [28]. Cooling rate in Mr. Frosty containers (1 °C/min) is faster than in the programmable freezer (0.17 °C/min), and too-fast cooling may not only cause cryoinjury due to formation of intracellular ice [40] but also function as a more powerful stressor inducing telomere shortening. In the ETs successfully recovered from cryopreservation, the telomeres were slightly longer than in non-cryopreserved ETs in the 2012 material, and slightly shorter in the 2014 material, but these differences were not significant. Thus, it can be concluded that successful cryopreservation does not affect telomere length in Norway spruce ETs.

To summarize the outcomes of the present study: SE initiation treatment containing several stress factors seems to cause telomere shortening in Norway spruce, and higher SE initiation frequencies in some families may relate to them having higher proportion of genotypes with longer telomeres. Following the initiation phase the telomere length in the induced ETs and mature embryos originating from them remains unchanged up to one year of culture, with remarkable genotypic variation observed also within the family. Prolonged in vitro culture can, however, shorten the telomeres significantly, and should be avoided. This can be achieved by cryopreservation of ETs, with successful cryopreservation treatment preserving telomere length. Somatic embryo production capacity of the ETs was observed to vary a lot not only among the genotypes, but also from one timepoint to another. No connection between embryo production numbers and the length of the telomeres was found, so this variation remains unexplained.

## 4. Materials and Methods

### 4.1. Plant Material

Embryogenic lines of Norway spruce were initiated from immature zygotic embryos originating in controlled crosses among the selected trees of the Finnish tree breeding programme. The crosses were made in 2012 and 2014 using grafts situated in southern Finland (2012: 60°39′ N, 24°01′ E, 60°55′ N, 26°13′ E, 2014: 60°41′ N, 24°02′ E), with both the mother trees and pollen donors originating from southern or central Finland. Immature green cones were collected for explant excision when the heat sum was around 800 d.d., i.e., approximately 10 weeks after pollination when the zygotic embryos had reached cotyledonary stage. Initiation of SE was performed with isolated zygotic embryos as explants as described by Varis et al. [28], using modified Litvay’s medium, mLM [41], containing half-strength macroelements and 10 mM 2,4-dichlorophenoxyacetic acid (2,4-D) and 5 mM 6-benzyladenine (BA) as plant growth regulators. In order to have material with broad genetic background, altogether 33 embryogenic lines representing 23 families (=controlled crosses) were selected for the study (Table A2 and Table A3). In addition, pooled samples of immature zygotic embryos (ZE) were prepared from eight crosses: At the time of SE initiation, part of the cones collected for SE explant preparation were put in cold-storage (+3 °C) until used. The ZE were then excised from immature seeds extracted from cold-stored cones, pooled cross-wise and frozen in liquid nitrogen.

### 4.2. Maintenance and Cryopreservation of Embryogenic Lines

The established embryogenic lines were subcultured every two weeks on the same mLM-medium as used for initiation. SE initiation success for each controlled cross was calculated as percentage of established embryogenic lines: (ZE explants developed into proliferating embryogenic tissue (ET)/all ZE explants) × 100. For further experiments, embryogenic tissue (ET) was collected 5–7 days following the last subculture.

Cryopreservation of ETs lines originating in 2014 crosses was performed approximately four months after their initiation, using a slow-cooling method [28]: Fresh growths from ETs were used as samples, and they were pretreated on semi-solid mLM media with increasing sucrose concentration (0.1 M for 24 h; 0.2 M for another 24 h). A mixture of polyethylene glycol 6000, glucose, and dimethylsulfoxide, 10% *w*/*v* each (PGD), was used as cryoprotectant, and the samples were frozen in a programmable freezer with a slow cooling rate (0.17 °C/min) to −38 °C, before immersing them in liquid nitrogen (LN).

ETs originating in 2012 crosses were older, approximately 14 months, at the time of cryopreservation. They were cryopreserved using the same method as 2014 material, but in the case of five lines, also using alternative methods, as described by Varis et al. [28]: Either the pretreatment on semi-solid media with increasing sucrose concentration and usage of the PGD cryoprotective mixture was combined with 2h-freezing in Mr. Frosty containers at −80 °C (lines 4934, 6375, 4611, 3128) or the ETs were pretreated for 24 h in liquid mLM medium supplemented with 0.4 M sorbitol, and dimethylsulfoxide (Me_2_SO) was added as a sole cryoprotectant to give the final Me2SO concentration of 10% (*v*/*v*), followed by freezing in a programmable freezer as described above (line 5852).

Following 1–2 months storage in LN, the samples were thawed in a water bath +37 °C 2 min and proliferation of ETs continued as described by Varis et al. [28]: The cryostorage liquid was drained off, and the ET washed with liquid mLM medium. The samples pretreated on the semi-solid media were placed on mLM medium with sucrose content of 0.2 M and transferred every 24 h on media with decreasing sucrose concentration (0.1 M and 0.03 M). Samples pretreated in liquid medium were placed on mLM medium with 0.03 M sucrose concentration and transferred to new media after 24 h. All ETs were then transferred to fresh mLM medium every two weeks.

### 4.3. Evaluation of Somatic Embryo Production Capacity

To test somatic embryo production capacity of the different lines at various time points, the filter maturation method modified from [42] and described by Varis et al. [28] was applied: About 180 (±20) mg of ET was mixed in 3 mL liquid mLM without plant growth regulators (PGR), and the suspension was poured onto paper filter (Whatman #2) placed in the Buchner funnel. The liquid was drained by suction and the filter was placed on mLM medium with 60 µM abscisic acid (ABA) and 0.2 M sucrose, gelled with 6 g/l of Phytagel. After eight weeks, the number of cotyledonary embryos per gram of fresh weight was counted for three dishes per line.

### 4.4. DNA Extraction and Southern Blot Analysis of Telomeric Repeats

Immediately after collection, the 300–500-mg samples of the ETs, mature somatic embryos or immature ZE were frozen in plastic bags in liquid nitrogen and stored at −80 °C. Total genomic DNA was isolated from the samples by the modified method of Lodhi et al. [43], as described [44,45]. DNA analysis was performed using Southern blot hybridization, as described by Kilian et al. [14], with the modifications described by Aronen and Ryynänen [9,10]. For the positive control, a synthetic telomere sequence was generated by PCR according to Cox et al. [46], using oligomer primers T1 (5′-TTTAGGG-3′) and T2 (5′-CCCTAAA-3′). Chemiluminescence detection of the hybridization products was performed according to the manufacturer’s (Roche Diagnostics GmbH) instructions. The output was then scanned with the AlphaImager Imaging System (Alpha Innotech Co./ProteinSimple, San Jose, CA, USA), and the size of the signals was analyzed using AlphaEase^®^FC software and digoxigenin labeled marker for molecular weight (MW). Only high molecular weight signals representing true telomeres at the end of the chromosomes were analyzed, i.e., clearly separate low molecular weight signals originating from centromeric or interstitial repeats observed in conifers [9] were not measured.

### 4.5. Experimental Design and Statistical Analyses

The effects of cryopreservation and aging of the ETs on the capacity of somatic embryo production were studied in 2014 material, together with genotypic variation. Somatic embryo maturation experiments in three replicates were performed with 4-, 8-, and 12-month-old ETs from 21 genotypes, as well as with cryostored and thawed ETs of the same genotypes, aged for four months prior to cryopreservation and two months following it (Table A2). Telomere length measurements were performed for 11 of these genotypes: Samples were taken from 4-, 8-, and 12-month-old proliferating ETs from continuous subculture, from cryostored and thawed, proliferating ETs, as well as from mature somatic embryos derived from 4-, 8-, and 12-month-old ETs. As a comparison, the length of telomeric repeats was also measured from immature ZE from eight controlled cross, using pooled samples (Table A2).

The effect of aging of the ETs on telomere length was also examined with the 2012 material that had been maintained for longer time: Five genotypes were sampled following either 14 or 22 months of continuous proliferation (Table A2).

The effect of cryopreservation and genotype on length of telomeric repeats in proliferating ETs was studied also in another experiment performed with 2012 material. Samples of all the 12 genotypes were taken prior to and following cryopreservation and successful regeneration of ETs (Table A2). As a comparison, five genotypes were also sampled when recovery of ETs failed.

The factors affecting embryo production capacity, i.e., age, cryopreservation, and genotype of ETs, were studied by analysis of variance. Furthermore, the effect of cryopreservation, aging of the ETs, tissue type (proliferating ET versus mature somatic embryos versus immature zygotic embryo), and genotype on length of telomeric repeats was studied by analysis of variance. Post hoc comparisons among the group means, if necessary, were performed using the Student–Newman–Keuls multiple range test. Pearson correlation coefficient was used to measure associations between studied variables, such as embryo production capacity and telomere length, or SE initiation frequency and telomere length of ZE explants. All the statistical analyses were performed using the IBM SPSS^®^ statistics 22.0 software.

## Data Availability

The data presented in this study are available on request from the corresponding author.

## Appendix A

**Table A1:** Success of somatic embryogenesis (SE) initiation in the controlled crosses of Norway spruce used in the present study.

Year and Cross	Number of Explants	Initiation %
2014		
E18 × E2273	200	71.5
E18 × E436	201	67.2
E18 × E5535	200	50.5
E207 × E1373	202	72.8
E207 × E252	200	30.0
E2105 × E2283	200	57.0
E2105 × E3354	67	53.7
E212 × E54	200	63.5
E46 × E3222	400	61.0
E462 × E1369	200	36.5
E9 × E1361	200	77.0
E162 × E81	200	51.0
E212 × E1518	11	54.6
E81 × E3224	132	68.9
E162 × E2149	202	37.6
E1551 × E2229	99	67.7
E799 × E1366	46	93,48
2012		
E2515 × K805	41	95.1
E2853 × E231	143	95.8
E2853 × E330	135	68.9
E318 × E231	72	69.4
E318 × K805	329	61.4
E329 × K805	22	100.0
K264 × E231	68	89.7
K264 × E330	89	85.4

**Table A2:** Norway spruce materials used in somatic embryogenesis and telomere length studies: Controlled crosses from which the embryogenic tissues (ETs) were derived, and genotypes used to examine embryo production capacity and length of telomeric repeats in ETs of varying age, prior to and following cryopreservation.

	Norway Spruce Genotypes Used in			
Year and Cross	Embryo Production Experiments	Telomere Length Measurements		
2014	Proliferating ETs of 4, 8, or 12 months in age; cryostored proliferating ETs, 4 + 2 months in age	Proliferating ETs of 4, 8, or 12 months in age; cryostored proliferating ETs, 4 + 2 months in age	Mature somatic embryos originating in ETs of 4, 8, or 12 months in age	Immature zygotic embryos, excised at time of SE initiation
E18 × E2273				yes
E18 × E436	653			
E18 × E5535	872			
E207 × E1373				yes
E207 × E252	1206	1206	1206	
	1305			
E2105 × E2283	1548	1548	1548	yes
E2105 × E3354	1606			
	1607			
E212 × E54	2181			
E46 × E3222	5820	5820	5820	
	2833			
E462 × E1369	3022	3022	3022	yes
E9 × E1361	4027	4031	4031	yes
	4031			
E162 × E81	243	243	243	yes
	259	259	259	
E212 × E1518				yes
E81 × E3224	3620			yes
E162 × E2149	30			
E1551 × E2229	4623	4623	4623	
	4639	4639	4639	
E799 × E1366	5101	5101	5101	
	5109	5109	5109	

**Table A3:** Norway spruce materials used in somatic embryogenesis and telomere length studies: Controlled crosses from which the embryogenic tissues (ETs) were derived, and genotypes used to examine embryo production capacity and length of telomeric repeats in ETs of varying age, prior to and following cryopreservation.

	Norway Spruce Genotypes Used in		
Year and Cross	Telomere Length Measurements		
2012	Proliferating ETs of 14 months in age; Cryostored proliferating ETs, 14 + 1 months in age	Cryostored ETs showing no recovery	Proliferating ETs of 22 months in age
E2515 × K805	3011		
E2853 × E231	3128	3128	
	3129		
E2853 × E330	4310		4310
E318 × E231	4932	4934	4934
	4934		
E318 × K805	6375	6375	6375
E329 × K805	9130		9130
K264 × E231	4611	4611	4611
	9110		
K264 × E330	3604	5852	
	5852		
